# Targeting tyrosine-kinases and estrogen receptor abrogates resistance to endocrine therapy in breast cancer

**DOI:** 10.18632/oncotarget.2022

**Published:** 2014-05-27

**Authors:** Shuying Liu, Xiaolong Meng, Huiqin Chen, Wenbin Liu, Todd Miller, Mandi Murph, Yiling Lu, Fan Zhang, Mihai Gagea, Carlos L. Arteaga, Gordon B. Mills, Funda Meric-Bernstam, Ana M. González-Angulo

**Affiliations:** ^1^ Department of Breast Medical Oncology, The University of Texas, MD Anderson Cancer Center, Houston, TX; ^2^ Department of Bioinformatics and Computational Biology, The University of Texas, MD Anderson Cancer Center, Houston, TX; ^3^ Department of Pharmacology & Toxicology, Norris Cotton Cancer Center, Geisel School of Medicine at Dartmouth, Lebanon, NH; ^4^ Department of Cancer Biology, Vanderbilt-Ingram Cancer Center, Vanderbilt University, Nashville, TN; ^5^ University of Georgia College of Pharmacy, Athens, GA; ^6^ Department of Systems Biology, The University of Texas, MD Anderson Cancer Center, Houston, TX; ^7^ Department of Veterinary Medicine and Surgery, The University of Texas, MD Anderson Cancer Center, Houston, TX; ^8^ Breast Cancer Research Program, Vanderbilt-Ingram Cancer Center, Vanderbilt University, Nashville, TN; ^9^ Department of Medicine, Vanderbilt-Ingram Cancer Center, Vanderbilt University, Nashville, TN; ^10^ Department of Surgical Oncology, The University of Texas, MD Anderson Cancer Center, Houston, TX

**Keywords:** breast cancer, targeting therapy, dasatinib, fulvestrant, MK0646

## Abstract

Despite numerous therapies that effectively inhibit estrogen signaling in breast cancer, a significant proportion of patients with estrogen receptor (ER)-positive malignancy will succumb to their disease. Herein we demonstrate that long-term estrogen deprivation (LTED) therapy among ER-positive breast cancer cells results in the adaptive increase in ER expression and subsequent activation of multiple tyrosine kinases. Combination therapy with the ER down-regulator fulvestrant and dasatinib, a broad kinase inhibitor, exhibits synergistic activity against LTED cells, by reduction of cell proliferation, cell survival, cell invasion and mammary acinar formation. Screening kinase phosphorylation using protein arrays and functional proteomic analysis demonstrates that the combination of fulvestrant and dasatinib inhibits multiple tyrosine kinases and cancer-related pathways that are constitutively activated in LTED cells. Because LTED cells display increased insulin receptor (InsR)/insulin-like growth factor 1 receptor (IGF-1R) signaling, we added an ant-IGF-1 antibody to the combination with fulvestrant and dasatinib in an effort to further increase the inhibition. However, adding MK0646 only modestly increased the inhibition of cell growth in monolayer culture, but neither suppressed acinar formation nor inhibited cell migration in vitro and invasion in vivo. Therefore, combinations of fulvestrant and dasatinib, but not MK0646, may benefit patients with tyrosine-kinase-activated, endocrine therapy-resistant breast cancer.

## INTRODUCTION

ER-positive disease is the most common type of breast cancer, accounting for nearly 75% of all cases. Although there are numerous therapeutic options available for these patients, allowing for a variety of approaches designed to inhibit the ER or estradiol synthesis and even sequentially alternating drugs for long-term suppression of estrogen signaling [1-6], drug resistance still develops [7-11]. Thus, a significant portion of patients with ER-positive disease will develop resistance after an overwhelming initial response and succumb to resistant disease.

We and others proposed that acquired resistance is due to the adaptive enhancement of ER expression and/ or activation of growth factor receptors after anti-estrogen therapy, as well as through crosstalk between ER and growth factor receptor signaling pathways, resulting in ligand-independent ER activation [12-20]. We previously established long-term estrogen deprivation (LTED) in ER-positive breast cancer cell lines, which models endocrine therapy-resistant breast cancer [17]. Using these cell lines as models, we demonstrated enhancement of InsR/IGF-1R pathway in LTED ER-positive cells. Although inhibition of InsR and IGF-IR suppressed cell growth, the effect increased when combined with fulvestrant [17, 18], a second-line, pure ER antagonist that destroys the receptor without any agonist effect [21, 22]. However, this didn't inhibit the invasion of LTED cells [17-19].

Dasatinib, broad specific tyrosine kinase inhibitor (BMS-354825; Bristol-Myers Squibb), was initially isolated as a dual SRC/ABL inhibitor and is used for the treatment of imatinib-resistant chronic myeloid leukemia [23, 24]. Growing evidence suggests that dasatinib not only targets ABL and the SRC family, but also targets multiple receptor tyrosine kinases (RTKs) and non-receptor tyrosine kinases. Through this activity dasatinib can inhibit cell proliferation, migration and invasion. Thus, it is currently being evaluated for use in multiple solid tumors [25].

The purpose of the present study was to seek a combination to successfully inhibit resistance pathways stimulated among ER-positive tumors. Thus, herein we evaluate the effect of combining fulvestrant with dasatinib and/or MK0646, an antibody that was developed as IGF1R antibody. We demonstrate that acquired resistance to LTED in ER-positive breast cancer is abrogated by combination of fulvestrant and dasatinib, which together inhibits multiple biological phenotypes, including acinar formation, cell survival and invasion as well as cell proliferation in vitro and vivo. However, adding MK0646 only modestly inhibited tumor cell growth, but didn't inhibit acinar formation, migration and invasion. The results of our study suggest that the combination of fulvestrant and dasatinib may be promising for the treatment of ER-positive disease that is resistant to prior hormonal therapy.

## RESULTS

### Long-term estrogen-deprived breast cancer cells are biologically more aggressive than their parental controls

**Figure 1:**
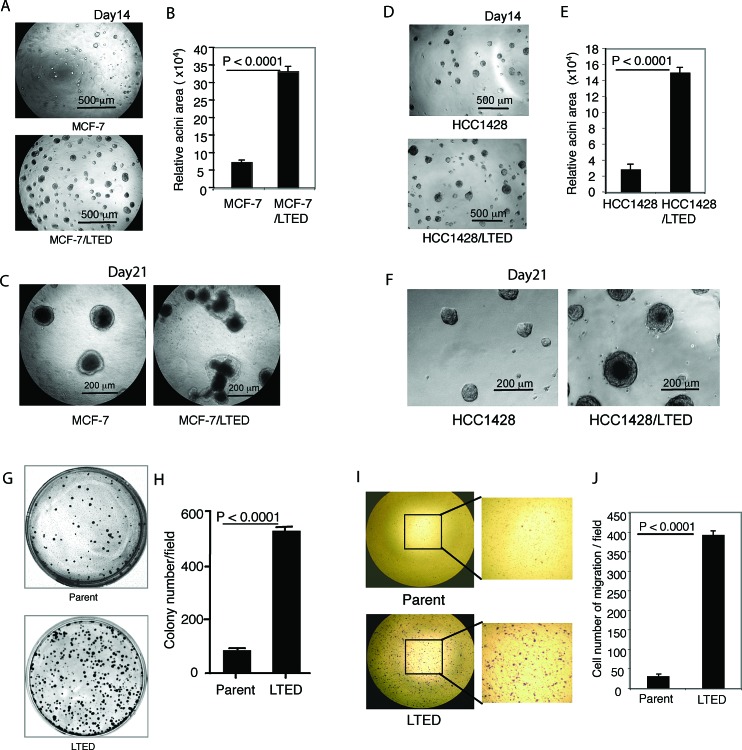
Characterization of LTED cells Parental and LTED MCF-7 and HCC-1428 cells were cultured on a bed of matrigel as described in Materials and Methods. Representative field images of acini were taken on day 14 (A, D), and taken on day 21 (C, F). The quantitative analysis of the total acinar area per field was performed with AlphaVIEW SA software. The data are mean +/− standard errors of triplicates, representative of two independent experiments (B, E). Clonegenic assay was performed as described in Materials and Methods. Parental and LTED MCF-7 ells were seeded at 1,000 cells in 60 mm dish in triplicates with growth medium for 13 days. The dishes were scanned (G) and quantitative analysis of the total number of clones was performed with AlphaVIEW SA software. The data are mean +/− standard errors of triplicates, representative of two independent experiments (H). I. Migration assay was performed as described in Materials and Methods. Parental and LTED MCF-7 cells that penetrated through pores to the underside of the filter were photographed and counted in 10 random fields. The data are mean+/− standard errors of triplicates, representative of two independent experiments (J).

Since we previously established ER-positive breast cancer cell lines after LTED [17], we sought to further understand their properties, thus we systematically evaluated their biological phenotypes. Mammary acinar formation assay showed that MCF-7/LTED cells formed numerous acini much larger than parental cells (*P* < 0.0001, Fig. 1A, B). When the 3-D culture was extended to 21 days, MCF-7/LTED cells formed highly proliferative, abnormal structures. Intriguingly, many cells spread into the surrounding matrigel of acini, suggesting the cells possess higher migration/invasion ability. In contrast, parental cells formed acini with a ‘normal’, more symmetrical, phenotype absent of spreading (Fig. 1C). These findings were recapitulated in HCC-1428/LTED cells (Fig. 1D-F). Clonogenic and migration assays using parental and LTED MCF7 cells demonstrated that LTED cells formed more numerous foci than parental cells (*P* < 0.0001, Fig. 1G, H). In addition, LTED cells showed markedly higher migration capability than their parental controls (*P* < 0.0001, Fig. 1I, J)

### LTED cells exhibit activation of multiple receptor tyrosine kinases and cancer-related pathways

Our previous work shows that ER-positive LTED cells display increased phosphorylation of InsR and IGF1R, whereas ER-negative LTED cells display increased phosphorylation of ErbB family members, and that combined inhibition of IGF1R and ErbB2 reduces cell proliferation over monotherapy [17]. Since these targeting therapies had no effect on cell invasion and metastasis remains a formidable clinical challenge, evading cure, we sought to find more efficient therapies targeting cell invasion. This is a fundamental experimental question since traditional chemotherapy can eliminate rapidly proliferating cancer cells but does not specifically target metastatic processes.

**Figure 2:**
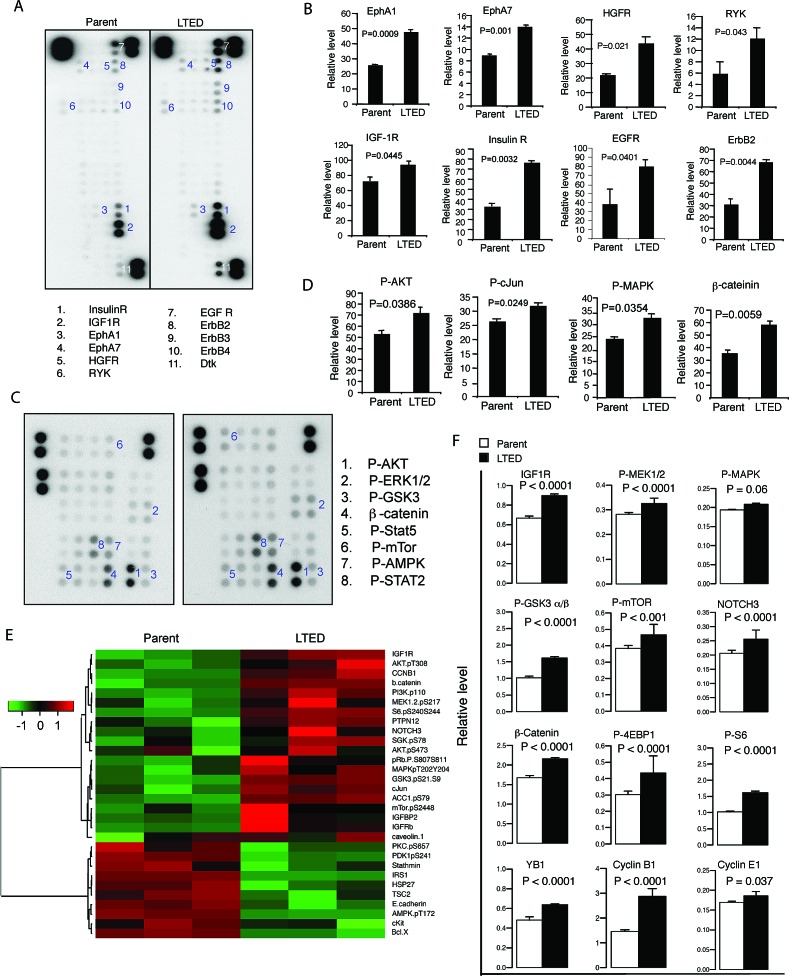
Signaling signature in LTED cells Phospho-RTKs (A, B) and their downstream kinase (C, D) were probed with lysates (300 μg per sample) from parental and LTED MCF-7 cells that stimulated with 10% DCC-FBS with IGF and EGF (10 ng/ml of each) for 10 minutes after starvation for overnight. Signal indicates phosphorylation of RTKs and downstream molecules. Corner spots are positive controls. Each sample was in duplicate (A, C). The signals were quantified by using AlphaVIEW SA analysis software. The data are mean +/− standard deviation (B, D). E. Parental and LTED MCF-7 cells in triplicates were starved for overnight, following by stimulation without or with 5% FBS for 30 minutes. The cell lysates were used for RPPA as described in Materials and Methods. Data are presented in a matrix format: each row represents an antibody target, and each column a sample. In each sample, the ratio of the abundance of the molecule to its median abundance across all samples is represented by the color of the corresponding cell in the matrix (see scale, for expression levels). F. The RPPA data was further analyzed described in Materials and Methods.

To investigate invasion, we began by studying signaling alterations of LTED cells using protein array and reverse-phase protein array. After screening 49 phosphorylated human RTKs and 43 downstream kinases using protein array (see Materials and Methods), we demonstrated enhanced activation of multiple RTKs, indicated by increased phosphorylation compared to parental cells, including ephrin receptors (EphA1, *P* = 0.0009; EphA7, *P* = 0.001), hepatocyte growth factor (HGFR) receptor (*P* = 0.021), receptor-like tyrosine kinase (RYK, *P* = 0.043) and developmental tyrosine kinase (Dtk, *P* = 0.001). We also demonstrated that LTED cells exhibited a higher response to IGF and EGF (10 ng/ml of each) than the parental cells, indicated by greater phosphorylation of IGF-1R, InsR (*P* = 0.0445, 0.0032, respectively) and ErbB receptors, including EGFR, ErbB2, ErbB3 and ErbB4 (*P* = 0.04, 0.004, 0.0027, 0.0189, respectively) (Fig. 2A, B and data not shown). Consistently, phosphorylation of their downstream kinases increased, including AKT, GSK3, c-Jun and MAPK as well as enhanced level of β-catenin (*P* = 0.0386, 0.0388, 0.0249, 0.0354, 0.0059 respectively) (Fig. 2C, D and data not shown). We further verified the findings using RPPA [26-28] (Fig. 2E). Consistent with the findings from protein array, LTED cells constitutively activated several cancer-associated pathways, including the PI3K-AKT and ERK/MAPK pathways, which in turn activate the effectors, including S6, 4EBP1, YB1, β-catenin, cyclin B1 (*P* < 0.0001 respectively) and cyclin E1 (*P* = 0.037, Fig. 2F). Therefore, our results led us to wonder whether inhibiting tyrosine kinases using the broad specific inhibitor dasatinib, and/or targeting InsR/IGF-1R pathway using the antibody MK0646, would synergize with the ER down-regulator, fulvestrant.

### Basis for combination therapy using fulvestrant, dasatinib and MK0646 on LTED cells

In our previous study, we found that ER expression adaptively increased in LTED ER-positive cells [17]. In the present study, we confirmed these findings using MCF-7/LTED and HCC-1428/LTED cells and also found that low dose fulvestrant (2 nM) markedly down-regulated ER expression (Supplementary Fig. S1A) and suppressed proliferation dose-dependently in both parental and LTED MCF-7 and HCC1428 cells, although response of LTED cells is lower than that of parental controls (Supplementary Fig. S1 B, C). To determine whether dasatinib effects cell signaling in LTED cells, we treated the cells with dasatinib (20 nM) and scanned 49 phosphorylated human RTKs using a protein array (see Materials and Methods). We observed that dasatinib inhibits multiple RTKs, including ephrin receptors (EphA1, *P* = 0.0067; EphA7, *P* = 0.0171; EphB2, *P* = 0.0078), HGFR (*P* = 0.0026), platelet-derived growth factor receptors (PDGFR) α/β (*P* = 0.0126, 0.015 respectively), RYK (*P* = 0.0108), and Trk tyrosine kinase receptors (TrkA, *P* = 0.036; TrkC, *P* = 0.0149) (Supplementary Fig. S1. D, E). Using MK0646, an antibody developed against IGF-1R, we demonstrated that MK0646 (100 μg/ml) almost completely inhibited IGF1R (*P*=0.0002). Strikingly, it not only restrained IGF1R, but also inhibited more than 70% InsR (*P*=0.0007, Supplementary Fig. S1 (F, G), which might be with more effective than targeting IGF-IR alone. Taking together, fulvestrant, dasatinib, and MK0646 potentially work together to treat acquired resistance to LTED in ER-positive breast cancer cells.

### Effects of combinations of fulvestrant, dasatinib and/or MK0646 on cell proliferation and survival

**Figure 3:**
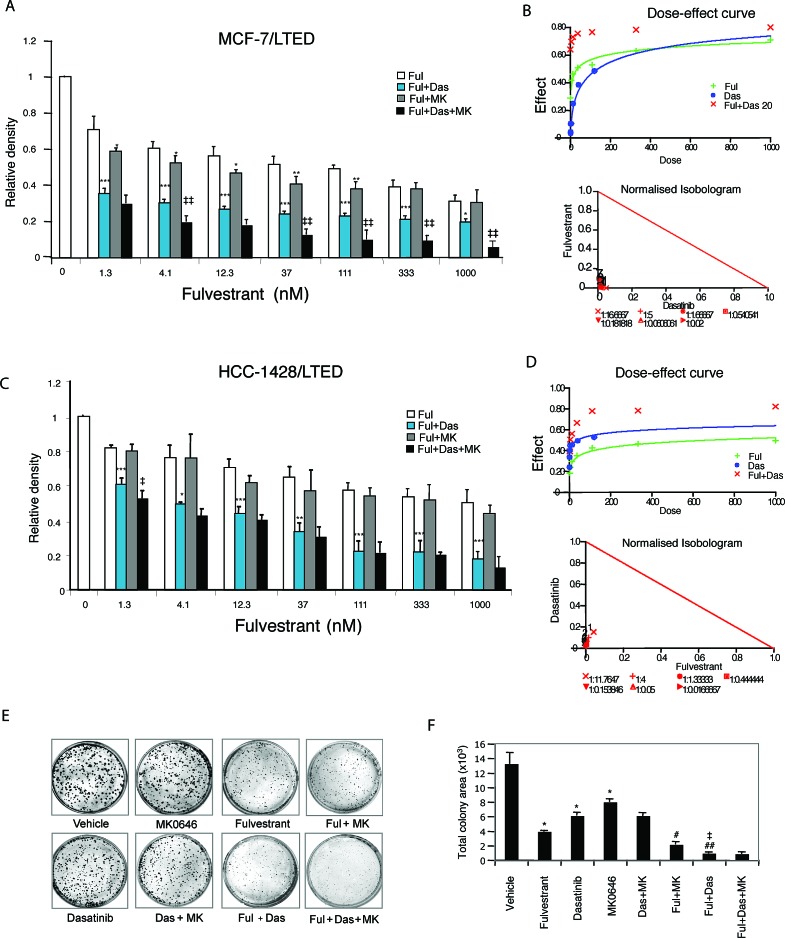
Effects of fulvestrant, dasatinib and/or MK0646 on LTED cell growth and survival LTED MCF-7 (A) and HCC1428 (C) cells were treated with fulvestrant alone in variable concentrations as indicated or addition of dasatinib (Das, 20 nM) and/or MK0646 (MK, 100 μg/ml) for 48 hours. Growth inhibition was determined using the CellTiter-Blue viability assay as described in Materials and Methods. Results of cell viability were calculated on the basis of percentage change to medium control containing vehicle. The data are mean +/− standard errors of triplicates, representative of two independent experiments [* P < 0.05, ** P < 0.01, *** P < 0.001 vs fulvestrant (Ful); ‡ P < 0.05, ‡‡ P < 0.01 vs combination of fulvestrant and dasatinib (Ful+Das)], ANOVA. Dose response curves and synergy analyses were generated for fulvestrant and dasatinib in MCF-7/LTED cells (B) and in HCC1428/LTED cells (D) using CalcuSyn Dose Effect Analyzer (detail see Materials and Methods). E. Clonegenic assay was performed as described in Materials and Methods. Quantitative analysis of number of foci (F) and total area of foci (G) was performed with AlphaVIEW SA software. The data are mean +/− standard errors of triplicates, representative of two independent experiments (* P < 0.0001 vs vehicle; # P < 0.05, ## P < 0.01 vs fulvestrant; ‡ P < 0.0001 vs dasatinib) ANOVA

To explore whether combinations of fulvestrant, dasatinib and/or MK0646 would increase therapeutic efficacy on cell proliferation and survival, we treated LTED cells in monolayer culture. In our preliminary studies, we detected the effect of each drug with variable doses. Fulvestrant (2 nM), dasatinib (20 nM), MK0646 (100 μg/ml) were used as fixed doses, and combined with each of the other two drugs in variable doses. The combination of fulvestrant and dasatinib significantly inhibited growth of MCF-7/LTED (*P* < 0.001, Fig. 3A). Synergy analyses exhibited synergistic interactions. B). These findings were recapitulated in HCC-1428/LTED cells (Fig. 3C, D). The combination of fulvestrant/MK0646 modestly decreased proliferation compared with fulvestrant alone in MCF-7/LTED (*P* < 0.05; Fig. 3A). Synergy analyses showed mild synergistic interaction when fulvestrant combined with high dose (100 μg/ml) MK0646 (Supplementary Fig. S2A) in MCF-7/LTED cells, but did not show the benefit in HCC-1428/LTED cells (Fig. 3C, Supplementary Fig. S2B). Adding MK0646 increased inhibitory effect of fulvestrant/dasatinib in MCF-7/LTED cells (*P* < 0.01; Fig. 3A), but didn't show further inhibitory effect in HCC1428/LTED cells (P > 0.05; Fig. 3C). Combination of high doses of dasatinib (20 nM or more) and MK0646 (100 μg/ml) mildly increased inhibitory effect compared with dasatinib or MK0646 alone in both MCF-7/LTED cells (Supplementary Fig. S2 C, D and data not shown) and HCC1428/LTED (Supplementary Fig. S2 E, F, data not shown). Taking together, combinations of fulvestrant/dasatinib markedly inhibit LTED cell proliferation; adding high dose of MK0646 (100 μg/ml) to fulvestrant or dasatinib mildly increased the inhibitory effect in both MCF-7/LTED and HCC1428/LTED cells. Addition of high dose MK0646 (100 μg/ml) to combination of fulvestrant/dasatinib further increased the inhibitory effect on MCF-7/LTED cells, but not on HCC1428/LTED cells.

To detect the effect of fulvestrant, dasatinib or MK0646 on cell survival, we performed clonogenic assay. MCF-7/LTED cells were treated with monotherapy or variable combinations as indicated (Fig. 3E, F, detail in Materials and Method). Comparing with vehicle, fulvestrant markedly decreased colony number and size (expressed as total colony area, *P* < 0.0001), whereas dasatinib and MK0646 had a lower effect than fulvestrant. Combination of dasatinib and fulvestrant further increased the inhibitory effect (*P* < 0.0001) than fulvestrant or dasatinib alone. Adding MK0646 to the combination of fulvestrant/dasatinib did not further increase the inhibitory effect.

### Addition of dasatinib, but not MK0646, to fulvestrant markedly inhibits mammary acinar formation and migration of LTED cells

**Figure 4:**
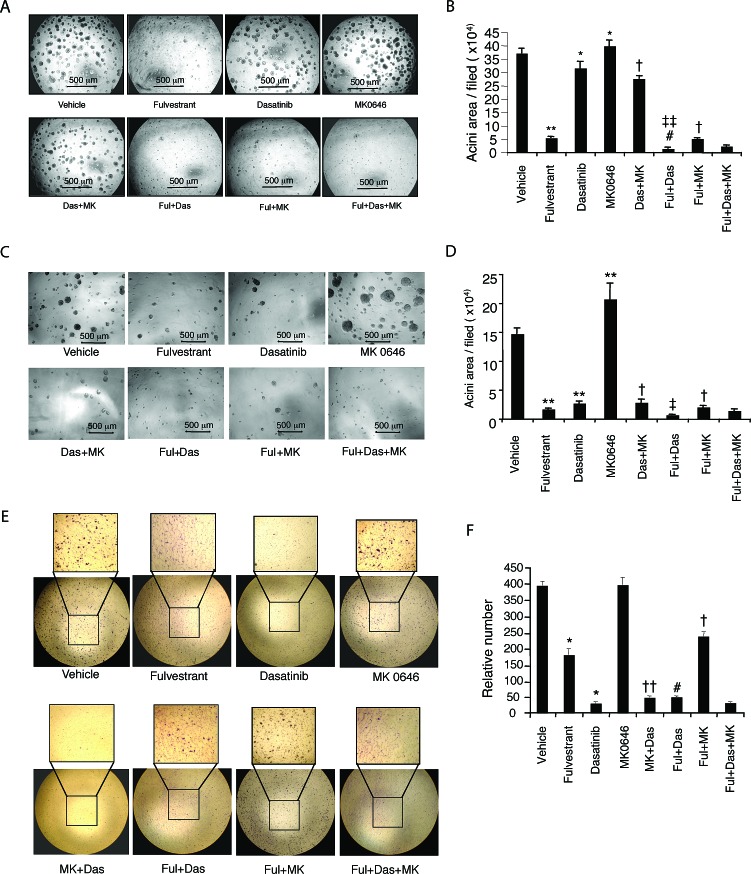
Effect of fulvestrant, dasatinib and/or MK0646 on mammary acinar morphogenesis and cell migration MCF-7/LTED and HCC-1428/LTED cells were cultured on a bed of matrigel with drugs in single (fulvestrant 2 nM, dasatinib 40 nM, MK0646 100 μg/ml) or combinations as indicated following the description in Materials and Method and Figure 1 legend. The medium with drugs was renewed every two days. Representative field images of MCF-7/LTED (A) and HCC1428/LTED (C) were taken on day 16. Quantitative analysis of total area of acini from MCF-7/LTED (B) or HCC1428/LTED (D) was performed with AlphaVIEW SA software. The data are mean +/− standard errors of triplicates, representative of two independent experiments (* *P* < 0.01, **, *P* <0.0001 vs Vehicle; # *P* < 0.05 vs fulvestrant (Ful); †, *P* <0.0001 vs MK0646 (MK); *‡ P* < 0.05, ‡‡ *P* < 0.0001 vs dasatinib (Das)] ANOVA. E. Effect of the drugs on cell migration was tested as described in Materials and Methods and legend of Figure 1. F. The data are mean+/− standard errors of triplicates, representative of two independent experiments (* *P* < 0.0001 vs vehicle; # *P* <0.0001 vs fulvestrant; †, P<0.001; ††, P<0.0001 vs MK0646) ANOVA.

Since LTED cells in 3-D culture exhibit increased proliferation and formation of abnormal acini structures compared with parental controls, we further explored the effects of fulvestrant, dasatinib and MK0646 as single agents or combinations on mammary acinar formation and morphogenesis. Our preliminary study showed that the dose of dasatinib (20 nM) used for the monolayer cell growth assay did not inhibit MCF-7/LTED cell growth in matrigel, hence we used 40 nM dasatinib as the fixed dose for combinations. Fulvestrant with low dose (2 nM) markedly, dasatinib (40 nM) mildly inhibited MCF-7/LTED cells growth in matrigel. Intriguling, MK0646 (100 μg/ml) didn't inhibit, but induced acini growth in both MCF-7/LTED (*P* < 0.01, Fig. 4A, B) and HCC1428/LTED cells (*P* < 0.0001, Fig. 4C, D). The addition of dasatinib to fulvestrant significantly inhibited acinar formation and proliferation (*P* < 0.0001), consistent with the effect on cell growth in monolayer cell culture. However, the addition of MK0646 to fulvestrant, or dasatinib or to the combination of fulvestrant/dasatinib did not further decrease the acini number or size in both MCF-7/LTED cells (Fig. 4A, B) and HCC1428/LTED cells (Fig. 4C, D).

We next studied the effects of the drugs on cell migration using transwells. Strikingly, we observed different patterns of drug effects on cell proliferation and acinar formation. In this regards, dasatinib markedly, and fulvestrant (4 nM) modestly, inhibited MCF-7/LTED cell migration induced by fibronectin (*P* < 0.0001, respectively). MK0646 did not show any inhibitory effect. Addition of fulvestrant and/or MK0646 to dasatinib did not further increase the inhibition over dasatinib alone (Fig. 4E, F).

### Combination of fulvestrant with dasatinib, but not MK0646, inhibits both tumor proliferation and invasion of LTED xenografts

**Figure 5:**
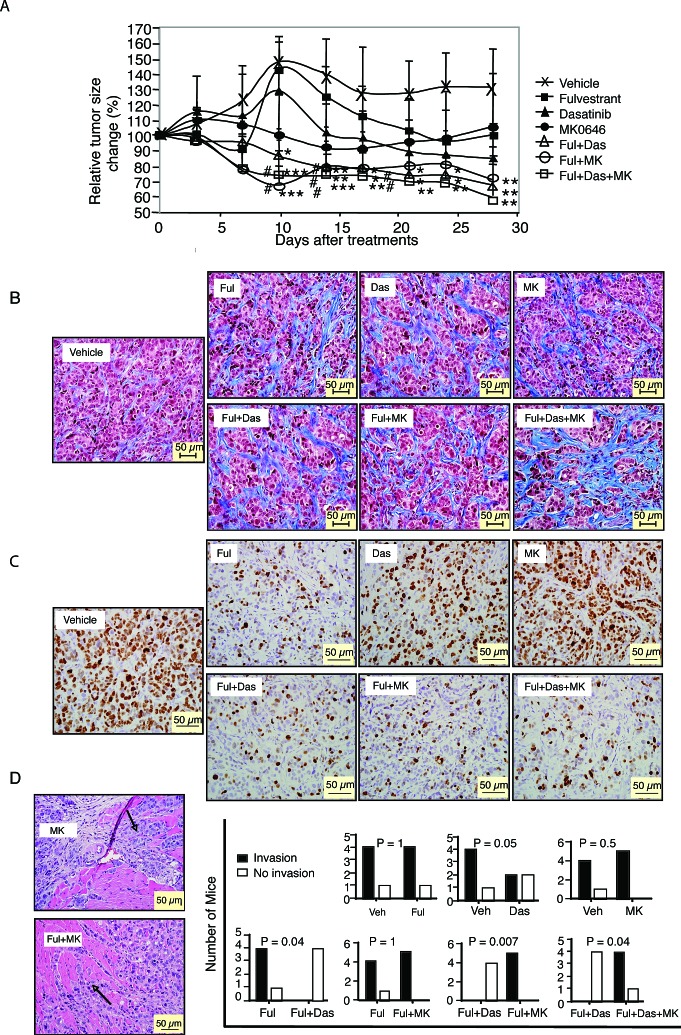
Effects of fulvestrant, dasatinib and/or MK0646 on MCF-7/LTED xenografts MCF-7/LTED cells were injected into mice without supplemented with E2. Seven days after tumor cell implantation, 100% (40/40) of the mice were burdened with tumor. The mice were randomized to the indicated treatments as described in Materials and Methods. One mouse of dasatinib (Das) group and combination of fulvestrant and dasatinib (Ful+Das) group died of gavage accidentally before the end of the study, so n = 4 in Das group and Das+Ful group; n = 5 in other groups. Tumor growth was plotted as the percentage of change in mean tumor volume/animal compared with day 0 of treatment indicated (A, * *P* < 0.05, ** *P* < 0.01; *** *P* < 0.001 versus vehicle; # *P* < 0.05; ## *P* < 0.01 versus fulvestrant) ANOVA. B. Masson's Trichrome staining for fibrosis of xenografts. C. Ki67 expression in xenografts. D. H&E for histological images from representative tumor from MK0646 (MK) or combination of fulvestrant and MK0646 (Ful + MK) group show tumor invasion to adjacent structures (left). Data are presented as number of tumor invasion, Fisher's exact test (right).

Next, we determined whether the effects seen in our *in vitro* assays are reflected under *in vivo* conditions. For this purpose, we established MCF-7/LTED xenografts in ovariectomized nude athymic mice without E2 pellet supplementation. Seven days after tumor cell implantation, 100% (40/40) of the mice had palpable tumors. The mice were treated with vehicle, fulvestrant, dasatinib, MK0646, or combinations as indicated in Fig. 5A (see Materials and Methods). Tumors in mice treated with dasatinib or fulvestrant continued to grow for several days before stabilizing at or near the original tumor volume. In contrast, MK0646 treated tumors remained relatively constant in size throughout the time period. At the end of the 28-day study, tumors treated with fulvestrant, dasatinib or MK0646 were approximately the same size as at the initiation of the study, while the tumors treated with vehicle increased by about 35%. In contrast, the combinations (fulvestrant/dasatinib, fuvestrant/MK0646, fulvestrant/dasatinib/MK0646) induced tumor regression with essentially similar activity over the time period of analysis. The ability of the fulvestrant/dasatinib combination to decrease tumor volume appeared delayed compared with the other combinations. On day 28^th^ of therapy, the mice were sacrificed. Xenograft tumors and all organs of each mouse were subjected to blinded histopathological analysis by a veterinary pathologist. Fulvestant, MK0646, or dasatinib alone mildly altered stromal content with increasing fibrosis formation (35%, 28%, 30% respectively), higher than that of vehicle group. Adding MK0646 to fulvestrant didn't increase fibrosis formation (30%). However adding dasatinb to fulvestrant or fulvestrant/MK0646 further increased stromal content (40%, 45%, respectively) (Fig. 5B). Fulvestrant markedly (70%) and dasatinib mildly (22%), decreased the level of Ki67 (the proliferation maker), while MK0646 did not alter Ki67 level compared with vehicle. Addition dasatinib and/or MK0646 to fulvestrant did not further increase the inhibitory effect of fulvestrant on Ki67 (73%, 70%, 72%, respectively) (Fig. 5C). Four of five tumors in vehicle group showed invasion to adjacent structures. Fulvestrant alone did not change the frequency of invasive structures. Dasatinib monotherapy decreased invasion, however, this failed to reach significance likely due to the small sample size.

Strikingly, the combination of dasatinib and fulvestrant completely blocked tumor invasion (*P* < 0.05). However, all tumors treated with MK0646 alone or combination of MK0646/fulvestrant exhibited invasion (Fig. 5D). There are few reports of distant metastatic disease in primary MCF-7 xenografts in the literatures limited that we know. In the MCF-7/LTED xenograft model, 3 cases of distant metastatic disease were documented. It is interesting that two of the three cases were in mice treated with MK0646: a metastatic adrenal gland tumor in the fulvestrant/MK0646 group (Supplementary Fig. S3A), and a metastatic tumor in a lymph node adjacent to the subcutaneous tumor in the MK0646 alone group (Supplementary Fig. S3B). There was one case of a lung intravascular tumor in the vehicle group (data not shown). However, no metastatic disease was found in mice treated with fulvestrant, dasatinib or fulvestrant/dasatinib, although the number of animals in each group was small.

### Effects of the combination of fulvestrant, dasatinib and/or MK0646 on cellular signaling in LTED cells

**Figure 6:**
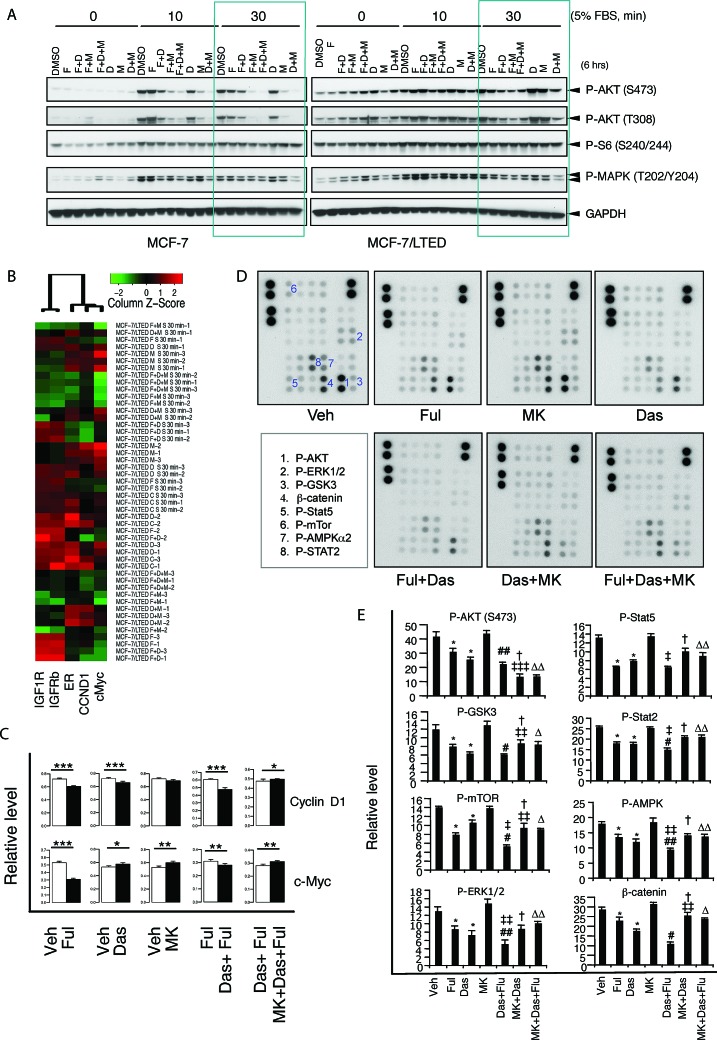
Combination of fulvestrant and dasatinib, but not MK0646, inhibits key effectors of cancer-associated signaling pathways LTED MCF-7 cells were starved for overnight, followed by treatment with fulvestrant (2 nM), dasatinib (20 nM), MK0646 (100 μg/ml) or combinations as indicated for 6 hours, following by stimulation with or without 5% FBS for variable time as indicated. The cell lysates were used for Western blot (A) and were further analyzed by RPPA (detail see Materials and Methods and the legend of Supplementary Fig. S4B). B. A sub-cluster of Supplementary Fig. S4B shows effects of the targeting therapy on ERα, IGFR, c-Myc and cyclin D1. C. The RPPA data of c-Myc and cyclin D1 was further analyzed described in Materials and Methods (* P < 0.05, ** P < 0.01, *** P < 0.001, **** P < 0.0001). D. MCF-7/LTED cells were treated with fulvestrant, dasatinib, MK0646 in single or variable combinations (as indicated) for 6 hours followed by stimulation with 10% DCC-FBS with 10 ng/ml IGF and 10 ng/ml EGF for 10 minutes. Cell lysates used for detecting phospho-kinases and expression of β-catenin(described in Materials and Methods and the legend of Figure 2).E. The signals were quantified by using AlphaVIEW SA analysis software. The data are mean +/− standard deviation. [*P < 0.0001 vs vehicle; # *P* < 0.05, ##, *P* < 0.0001 vs fulvestrant; ‡ *P* < 0.05, *P* < 0.01, P < 0.0001 vs Das; †, *P* < 0.0001 vs MK0646; ∆ *P* < 0.001, ∆∆ *P* < 0.0001 vs combination of fulvestrant and dasatinib (Ful+Das)] ANOWA.

To identify the mechanism by which the combination of fulvestrant and dasatinib abrogates resistance to endocrine therapy in breast cancer, we systematically detected effects of the targeting therapies on cancer-related signaling that elevated in LTED cells using Western blot, protein array and reverse phase protein array. Parental and LTED MCF-7 and HCC1428 cells were treated for 6 hours with fulvestrant, dasatinib or MK0646 either as single agents or in variable combinations followed by stimulation with or without 5% FBS in variable time. The cell lysates were analyzed with Western blot for phosphorylation of AKT, MAPK and S6, the key molecules in PI3K-AKT and ERK/MAPK pathways (Fig. 6A, Supplementary Fig. S4A). Compared with the parental cells, LTED cells exhibited elevation of phosphorylation of AKT, MAPK and S6 without FBS stimulation and stronger response to FBS-stimulation, suggesting constitutively activation of PI3K-AKT pathway and ERK/MAPK pathway, which is consistent with findings from RPPA and protein array showed in Fig. 2. Fulvestrant, dasatinib in single reagent didn't or mildly inhibited phosphorylation of AKT and MAPK in both parental and LTED cells. MK0646 monotherapy inhibited phosphorylation of AKT in parental cells, but not in LTED cells with FBS-stimulation. All combinations exhibited inhibitory effect in both parental and LTED cells in variable scale (Fig. 6A, green square, Supplementary Fig. S4A), suggesting combinations of fulvestrant, dasatinib or MK0646 effectively inhibit PI3K-AKT and MAPK pathways.

We further explored variation of expression or phosphorylation of 98 proteins in 32 experiment samples with biological triplicates (For a sample list, see Supplementary Table S1; Antibody list see Supplementary Table S2) by RPPA (Supplementary Fig. S4B). On the basis of unsupervised hierarchical clustering, parental cells without FBS stimulation form a cluster at the bottom of the dendrogram, followed by a cluster of parental cells stimulated with 5% FBS, and then LTED cells without FBS simulation. The upper cluster is formed by LTED stimulated with 5% FBS. Thus, in terms of protein and phospho-protein levels, LTED are distinct from parental cells with FBS altering protein and phospho-protein levels in both groups. The effects of the targeting therapies did not alter the composition of the main clusters formed by LTED and FBS stimulation, indicating that the LTED cells are markedly different from the parental cells. Interestingly MK0646 monotherapy resulted in MCF-7/LTED cells without FBS stimulation to share characteristics with MCF7/LTED cells stimulated with FBS (Pink frame in Supplementary Fig. S4B, red in Supplementary Table S1). As expected, fulvestrant markedly down regulated ER; MK0646 markedly inhibited both IGF-1R and IGFR-β (Fig. 6B), consistent with the findings from Western blot (Supplementary Figure S1A, F). Combination of fulvestrant and dasatinib significantly inhibited PI3K-AKT, ERK/MAPK pathways indicating by decreased phosphorylation of AKT, GSK3, MEK and MAPK, while adding MK0646 to the combination of fulvestrant/dasatinib didn't further increase the inhibitory effect (Supplementary Fig. S4B and data not shown). Consistently, the combination of fulvestrant and dasatinib decreased the key effectors including c-Myc and cyclin D1. However, addition of MK0646 abolished the inhibitory effects of fulvestrant or fulvestrant/dasatinib (Fig. 6B, C). Consistent with the findings from RPPA, protein array analysis exhibited that dasatinib markedly, fulvestrant mildly, decreased β-catenin (*P* < 0.0001), while MK0646 didn't show inhibitory effect. Combination of fulvestrant and dasatinib further increased the inhibitory effects. However, addition of MK0646 to dasatinib or to combination of fulvestrant/dasatinib abolished the inhibitory effect on β-catenin, even showed antagonistic (*P* < 0.01, 0.001 respectively). The effects of fulvestrant, dasatinib and MK0646 in single or combinations on phospho-type of Stat5, Stat2, AMPK, MAPK (ERK1/2), mTOR and GSK3 exhibited similar pattern with β-catenin, although addition of MK0646 to dasatinib or fulvestrant/dasatinib further increased the inhibitory effect on phospho-AKT (Fig. 6D, E).

## DISCUSSION

Herein, we demonstrate that LTED ER-positive breast cancer cells exhibit a more aggressive phenotype than primary controls, including increased cell proliferation, mammary acinar formation, cell survival and migration. We also show that the combination of fulvestrant and dasatinib inhibits tumor cell proliferation and suppresses cell survival and invasion *in vitro* and *in vivo*. An extrapolation of our results hints at the exciting potential that the combination of dasatinib and fulvestrant could benefit patients with ER-positive breast cancer that has become resistant to first-line endocrine therapy like tamoxifen or aromatase inhibitors.

**Figure 7:**
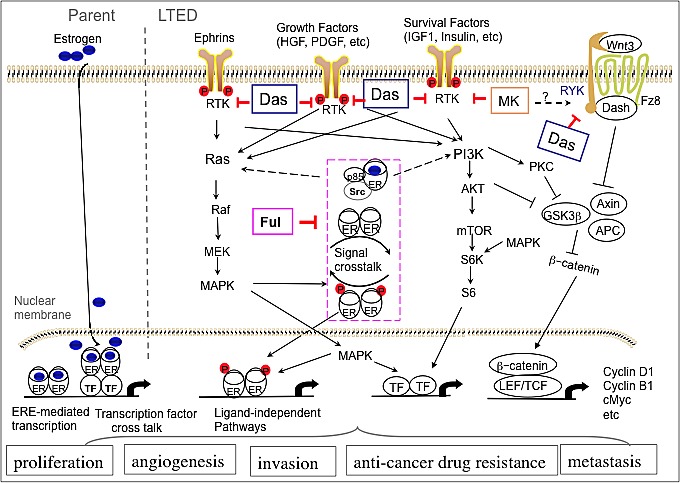
Mechanism by which targeting tyrosine-kinases and estrogen receptor overcomes endocrine-therapy resistance in Breast Cancer Beginning with phospho-RTK and other kinase screening and functional proteomic analysis with RPPA on LTED ER-positive breast cancer cells with or without treatment with fulvestrant, dasatinib or MK0646 and combining the published reports, a resultant pathway signature mediated by LTED and the targeting therapies is illustrated. After targeting estrogen therapy, small number of tumor cells acquired adaptive capacities, including: 1) compensatory increase ER Expression; 2) activation of multiple receptor tyrosine kinases; 3) signal crosstalk between ER and growth factor receptor pathways. Fulvestrant down-regulates ER and suppresses the signal crosstalk (pink frame). Dasatinib targets multiple RTKs, including EphAs, HGRR, RYK as well as non-receptor tyrosine kinases. Combination of fulvestrant and dasatinib inhibits cancer cell proliferation and invasion by blocking multiple receptor or non-receptor tyrosine kinase and targeting ER and crosstalk between ER and growth factor signaling. MK0646 targets IGF1R and Insulin R pathway, however, somehow activates RYK and Wnt pathway, leading β-catenin accumulation and relocates in nuclei, where, in turn, induce transcription of c-Myc and cyclin D1.

Based upon our data combined with published reports, we constructed a resultant pathway signature of LTED cells (Fig. 7). This graphic illustrates the mechanism by which LTED cells escape estrogen dependence, acquire a more aggressive biological phenotype and how fulvestrant and dasatinib interplay in this signaling pathway. In summary, LTED cells exhibit activation of multiple RTK: EphA1, EphA7, EphB2, HGFR, RYK, PDGFRα/β, TrkA and TrkC. They also show an enhanced response to IGF and EGF, which in turn activates PI3K-AKT and ERK/MAPK signaling pathways, which cross-talk with elevated ER. Combination of fulvestrant and dasatinib significantly inhibits PI3K-AKT, ERK/MAPK and Stat pathways – the central pathways regulating cancer cell proliferation, survival, invasion and drug-resistance. The signaling alterations of LTED cells in turn feed into multiple transcriptional mediators, including β-catenin, c-Myc, and cyclin D1, B1 and E1, all which are activated as a result of the activated signaling.

Our results regarding the activity of dasatinib are in slight contrast with a previous analysis. In a prior study, it was reported that dasatinib inhibited EGFR, HER-2 and HER-3 expression in breast cancer cells, specifically the cell lines MDA-MB-468, SKBR3, MDA-MB-453, and MDA-MB-231[29]. In our current study, dasatinib did not inhibit or mildly inhibited phosphorylation of ErbB family members. The reasons for this are not clear, although it may be the difference in cell lines between these studies. Therefore, the combination of an effective inhibitor for EGFR and HER2 with fulvestrant and dasatinib may further benefit patients with high ErbB family activity, endocrine therapy-resistance breast cancer.

In our previous study, we found that IGF1R/InsR inhibitor, AEW541, showed inhibitory effects on LTED ER-positive cell growth, whereas the neutralizing IGF-IR monoclonal antibody, MAB391, was ineffective, which was explained that therapeutic targeting of both InsR and IGF-IR should be more effective than targeting IGF-IR alone [19]. In the present study, we used another neutralizing monoclonal antibody, MK0646 that inhibits both IGF1R and InsR, and also found this to be ineffective overall, although in a few experiments the addition of MK0646 to fulvestrant and dasatinib mildly suppressed monolayer and decreased tumor size of xenografts. In this regards, adding MK0646 to fulvestrant or fulvestrant/dasatinib combinations did not increase the inhibitory effect on tumor cell migration, invasion or mammary acinar formation. Instead, MK0646 mostly abolished the inhibitory effects of fulvestrant/dasatinib combinations.

Several clinical trials evaluating MK0646 and other IGF1R monoclonal antibodies in patients with various types of solid tumors, including breast cancer are nearing a conclusion or already completed. It is not surprising given our observations that the early reports were at variance [30, 31], but mostly disappointing. It was previously unknown how the mechanism related to the results. In the present study, functional proteomic analyses allow us to demonstrate that although MK0646 blocked IGF-1R/InsR and inhibited some molecules of PI3K-AKT pathway. However, the addition of MK0646 abolished the inhibitory effect of fulvestrant or dasatinib/fulvestrant combinations on multiple molecules including Stat5 and Stat2, mTOR, MAPK and β-catenin, the key effector of Wnt pathway and elevated the key effectors, c-Myc and cyclin D1. Our RTK-screen showed that MK0646 increased the level of RYK (data not shown), the atypical receptor tyrosine kinase, that mediates Wnt signaling independently of Frizzled and/or function as a Frizzled co-receptor [32, 33], potentially increased β-catenin (Fig. 7). Although the mechanism by which MK0646 increase β-catenin, c-Myc and cyclin D1 remains unknown, our findings may help to explain why several clinical trials on IGF-1R monoclonal antibodies have been discontinued [34, 35]. We hope that our results may lead to develop new types of monoclonal antibody against IGF1R/InsR. For example, a “one-armed” monovalent antibody that prevents antibody-induced dimerization and results in potent antagonism [36] or the incorporation of a small molecular RTK inhibitor, such as OSI-906 or AEW541, targeting IGF-1R/InsR signaling instead of monoclonal antibodies.

Our results are significant in their implications for patient management. Although fulvestrant is a second-line therapeutic used to treat ER-positive breast cancer patients, it has limitations. Even though it effectively inhibits mammary acinar formation and cell proliferation, the ability of fulvestrant to inhibit cell migration and invasion is inadequate, and it has only a partial ability to induce cell death. Thus, additional therapeutics are needed to compensate for this imperfections. This is the rationale for our focus on new combination approaches. Hence, our demonstration that dasatinib inhibits multiple tyrosine kinases in LTED cells and also cell migration *in vitro* and invasion *in vivo*. In summary, dasatinib synergizes with fulvestrant to inhibit the growth of LTED cells. The combination of fulvestrant with dasatinib, but not MK0646 is effective in inhibiting acinar formation and tumor cell invasion, suggesting that the combination therapy of dasatinib and fulvestrant may be a therapeutic strategy for the anti-endocrine therapy resistant ER-retained breast cancers.

## MATERIALS AND METHODS

### Cell lines and culture conditions

Parental HCC-1428 and MCF-7 lines (ATCC) were maintained in IMEM supplemented with 10% fetal bovine serum (FBS, Gibco). HCC-1428/LTED and MCF-7/LTED cells were generated through culture in phenol red-free IMEM supplemented with 10% dextran-charcoal-treated FBS [DCC-FBS (Hyclone)] [17]. All media were supplemented with 100 units /ml penicillin, and 100 μg/ml streptomycin.

### Drugs

Dasatinib was purchased from Selleck Chemicals (Houston, TX). Fulvestrant (ICI 182,780) was purchased from Tocris Bioscience, (Ellisville, MO). For *in vitro* use, fulvestrant and dasatinib were dissolved in DMSO (Sigma-Aldrich) to a concentration of 1 mM respectively, stored at −20^o^C, and further diluted to an appropriate final concentration in serum-free medium upon use. DMSO in the final solution was 0.1% (v/v). For *in vivo* studies, dasatinib 50 mg/kg (in 50 μl of DMSO) was given daily by oral gavage. Fulvestrant (5 mg/50 μl DMSO/mouse, diluted with 50 μl peanut oil, subcutaneously) was administered weekly. MK0646, a humanized monoclonal antibody against IGF-1R was provided by Merck & Co. (Whitehouse Station, NJ) and used as per manufacturer's instructions. For *in vivo* studies, MK0646 (10 mg/kg, i.p.) was administered weekly.

### Cell growth inhibition assays

Cells were seeded in 96-well plates in growth medium and allowed to attach for 24 hours. Medium was changed to 2% FBS and cells were incubated at 37^o^C overnight, followed by the addition of serial dilutions of drugs. Growth inhibition was determined 48 hours later using the CellTiter-Blue viability assay according to manufacturer's protocol (Promega). Results of cell viability were calculated on the basis of percentage change versus vehicle-treated control. Drug concentrations for combinations were chosen in fixed-ratio increments. Dose-response curves were generated using CalcuSyn software (Biosoft, Ferguson, MO), following the manufacturer's guide. Combination Index (CI) < 1, =1, and >1 indicate synergism, additive, and antagonism, respectively.

### Clonogenic assay

To detect the effects of long-term estrogen deprivation on cell colony formation, MCF-7 and MCF-7/LTED cells were seeded at 1,000 cells in 60 mm dishes with growth medium for 13 days. For inhibitory assay, after having attached on the dish, MCF-7/LTED cells were treated for 48 hours with drugs in single or variable combinations as indicated. Then the drugs were washed away and cells were allowed to grow in growth media for 13 days. Dishes were scanned after staining. Quantitative analysis of the number and total area of clones were performed with AlphaVIEW SA software (Cell Biosciences). Data expressed as means of triplicates, representative of two independent experiments.

### Morphogenesis assay

Three-dimensional (3-D) culture of cells on matrigel basement membrane was carried out as described [37]. Briefly, 4 x10^3^ cells were resuspended in modified growth medium containing 2% growth factor-reduced matrigel (BD Biosciences) without or with drugs (2 nM fulvestrant, 20 nM Dasatinib, 100 μg/ml MK0646, or combinations) or vehicle DMSO, and seeded onto matrigel in 8-well chamber slides (BD Bioscience). Medium with drugs was replaced every 2 days. Photographs of representative fields were taken as indicated. Acini were photographed and counted in 10 randomly chosen fields. Quantitative analysis of the number and total area of acini were performed with AlphaVIEW SA software. Data expressed as means of triplicates, representative of two independent experiments.

### Migration assay

Cell migration assays were performed in 24-well chambers with 8 μm polycarbonate filters (Becton Dickinson). After starvation for 20 hours in serum-free IMEM, 1×10^5^ cells in 0.6 ml IMEM were placed in the upper chamber, and 0.75 ml of growth medium with fibronectin (5 μg/ml) as a chemo-attractant was added to the lower chamber. Drugs (fulvestrant 4 nM, dasatinib 40 nM, MK0646 100 μg/ml) or vehicle were added to both the upper and lower chambers. Cells were allowed to migrate through the filter at 37°C, 5% CO2 for 24 hours. Non-migrated cells on the upper surface of the filter were removed. The cells that penetrated through pores to the underside of the filter were stained, photographed and counted in 10 random fields. Data expressed as means of triplicates, representative of two independent experiments.

### Mouse xenografts and *in vivo* drug studies

Ovariectomized Balb/c athymic (*nu*/*nu*) mice were purchased from Charles River Laboratories International, Inc (Wilmington, MA) at 5 weeks of age and housed in sterile filter-capped cages. Animal studies were carried out under ACUF-approved protocols. Exponentially growing MCF-7/LTED cells were harvested, washed, and resuspended in PBS, and mixed with growth factor-reduced matrigel (1:1, Becton Dickinson). 10^7^ cells in 200 μl were injected into the mammary fat pads of mice at 6 weeks of age. Our preliminary studies demonstrated that MCF-7/LTED cells form tumors and sustain tumor growth for more than one month without estrogen supplementation. Primary MCF-7 cells require estrogen supplementation to maintain tumor growth [18, 38, 39](data not shown). When established tumors were detected (~7 days after cell injection), mice were randomized to treatment with vehicles, fulvestrant, dasatinib, MK0646, or variable combinations (n = 5/group). Tumor sizes were determined by measuring the length (*l*) and the width (*w*) with calipers twice weekly. Tumor volume was calculated with the formula (*V* = *l* × *w^2^*/2). Differences in tumor volume among treatment groups at each time point were analyzed using ANOVA. At the end of the experiment, mice were sacrificed. Tumors were harvested and flash-frozen in liquid nitrogen or fixed in 10% neutral-buffered formalin for paraffin embedding. Histological analysis of tumors and all organs were performed with hematoxylin-eosin. Tumor xenograft specimens were also stained with Masson's trichrome.

### Immunohistochemistry (IHC)

Paraffin sections were immunostained after antigen retrieval with boiling in 10 mM sodium citrate buffer for 20 minutes. The antibody to Ki67 (1:100; Abcam) was used at room temperature (RT) for 60 minutes, followed by incubation with Dako EnVision+ System-HRP Labeled Polymer (Anti-Rabbit). Throughout the above analyses, controls were prepared by omitting the primary antibody.

### Western blot

Western blot was performed as described [40]. Antibodies used here were also used for reverse phase protein array.

### Kinase screen with protein array

Phosphorylation of RTKs and other kinases was screened with protein array method using human phospho-RTK array kits and human phospho-kinase array kits (R&D Systems). Relative levels in cell lysates (300 μg per sample) were analyzed according to manufacturer's protocol and quantified with AlphaVIEW SA analysis software.

### Reverse phase protein array (RPPA)

Cell lysates used for Western blot were also used for RPPA. Samples are listed in Supplementary Table S1. RPPA was performed as previously described [26-28]. The antibodies used for RPPA are listed in Supplementary Table S2.

### Statistical analyses

Statistical analysis was carried out using ANOVA test (for multiple groups) and Student *t* test (for two groups). Fisher's exact test was used for analysis of tumor invasion data. Differences with *P* values of < 0.05 were considered statistically significant. Statistical analysis for RPPA data in Supplementary Methods.

## SUPPLEMENTARY METHODS FIGURES AND TABLES


