# Supplementary material for: Targeting tyrosine-kinases and estrogen receptor abrogates resistance to endocrine therapy in breast cancer
Source: Oncotarget. 2014 May 27;5(19):9049–64. doi: 10.18632/oncotarget.2022 (PMC4253418; doi:10.18632/oncotarget.2022)
Supplement: Supplementary file 1 [file oncotarget-05-9049-s001.pdf]

Targeting tyrosine-kinases and estrogen receptor abrogates resistance to endocrine therapy in breast cancer

Supplementary Methods

Statistical analysis for RPPA data

Images of RPPA slides were quantified by MicroVigene (Version 2.9.9.7, VigeneTech, Inc., Carlisle, MA). Spot signal intensity data were processed using the R package *SuperCurve* (Version 1.4.1) (33), available at http://bioinformatics.mdanderson.org/OOMPA/2.13. The data were normalized to correct the loading effect by the following: median-center raw protein concentration data (in log2 scale) for each protein; subtract the median protein concentration of each sample from the raw data of the sample. Hierarchical clustering of samples and proteins was performed using a distance matrix based on Pearson correlation coefficient $d=(1-r)/2$, where d is the distance between two samples or biomarkers, and r is the Pearson correlation coefficient) and Ward's minimum variance-based agglomeration. The R package *limma* (34) was used to fit a linear model for each protein, with the compound condition, cell type (parent or LTED) + stimulation status (serum or no serum) + treatment (drug or combinations) as the single-predicting variable. Thirty-four contrasts were constructed from each model. Q-values (35, 36) were calculated from the p-values obtained from the modified t-tests in *limma*. Significance calls were based on q-value < 0.05 and fold change > 1.5 or < 0.667 (1/1.5).

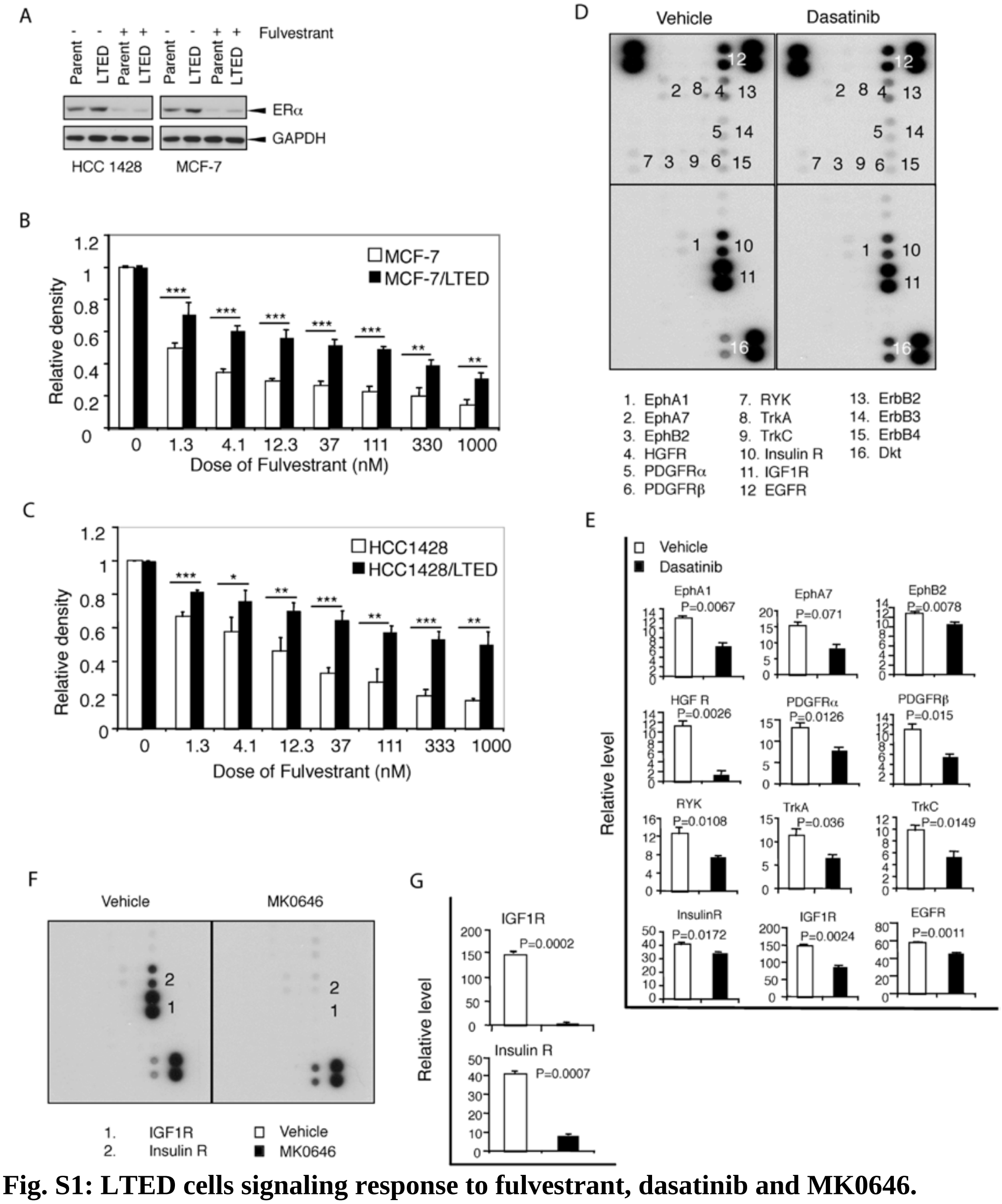

Fig. S1: LTED cells signaling response to fulvestrant, dasatinib and MK0646.

A. Parental and LTED MCF-7 and HCC1428 cells were treated without or with fulvestrant (2nM) for 6 hours. Cell lysates were used to detect ERα expression with Western blot, GAPDH as loading control. Parental and LTED MCF-7 cells (B) and HCC1428 cells (C) were treated with fulvestrant in indicated concentrations for 48 hours. Growth inhibition was determined using the CellTiter-Blue viability assay as described in Materials and Methods. Results of cell viability were calculated on the basis of percentage change to medium control containing vehicle. The data are mean +/- standard errors of triplicates, representative of two independent experiments (* $P < 0.05$, ** $P < 0.01$, *** $P <$

0.001) Student *t* test. LTED MCF-7 cells were treated with 20 nM dasatinib (D) or 100 µg/ml MK0646 (F) for 6 hours after starvation for overnight and followed by stimulation with 10% DCC-FBS with IGF and EGF (10 ng/ml of each) for 10 minutes. Cell lysates were used for detecting phosphorylation of RTKs (detail see Materials and Methods and the legend of Fig. 2). Corner spots are positive controls. Each sample was in duplicate. The signals were quantified by using AlphaVIEW SA analysis software. The data are mean +/- standard deviation (E,G). Student *t* test.

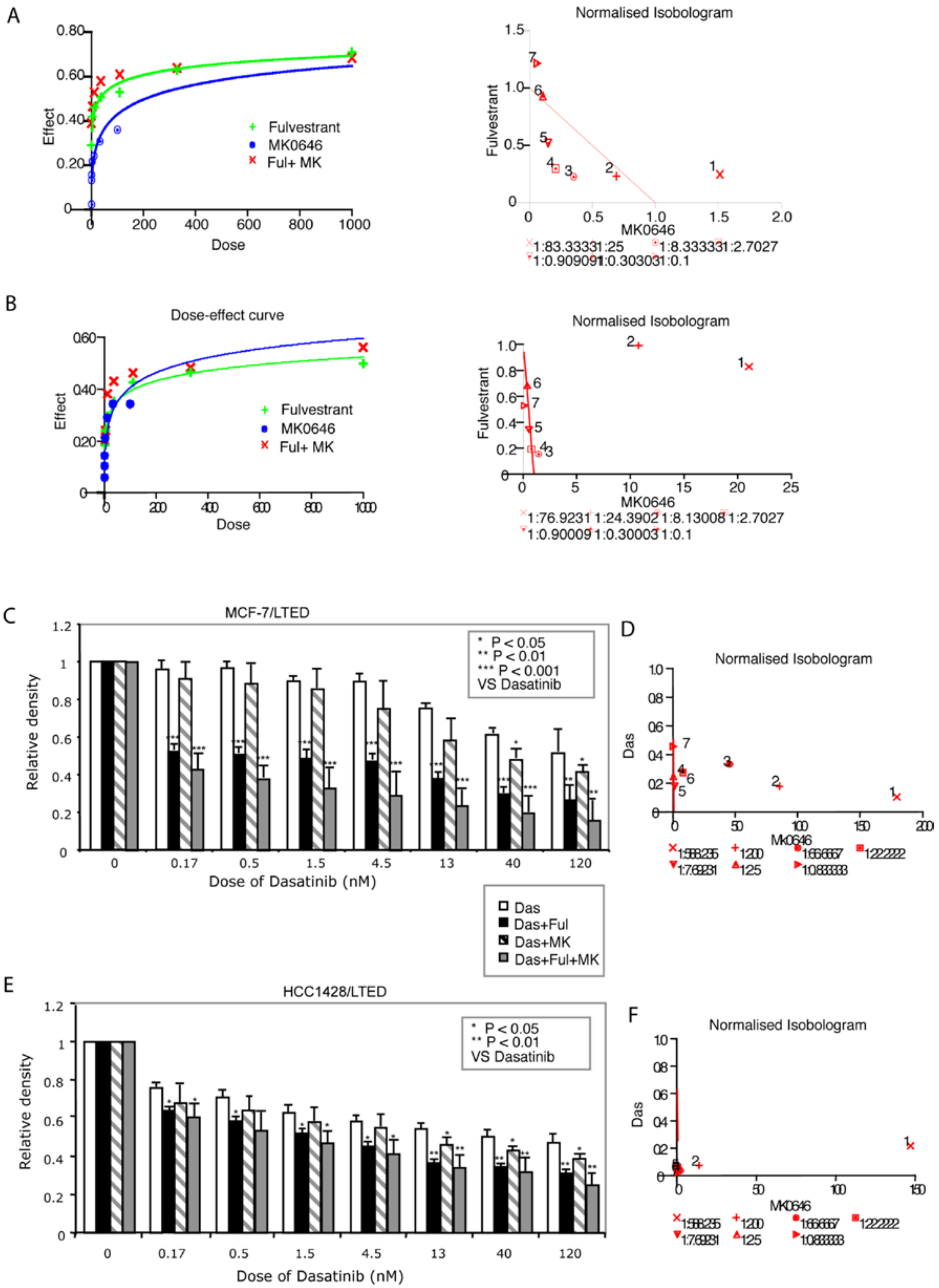
A
Effect
Dose
Fulvestrant
MK0646
Ful+ MK
Normalised Isobologram
Fulvestrant
MK0646
1:83.33331:25
1:8.333331:2.7027
1:0.909091:0.303031:0.1
B
Dose-effect curve
Effect
Dose
Fulvestrant
MK0646
Ful+ MK
Normalised Isobologram
Fulvestrant
MK0646
1:76.9231 1:24.3902 1:8.13008 1:2.7027
1:0.90009 1:0.30003 1:0.1
C
MCF-7/LTED
Relative density
Dose of Dasatinib (nM)
* P < 0.05
** P < 0.01
*** P < 0.001
VS Dasatinib
Das
Das+Ful
Das+MK
Das+Ful+MK
D
Normalised Isobologram
Das
Mk0646
E
HCC1428/LTED
Relative density
Dose of Dasatinib (nM)
* P < 0.05
** P < 0.01
VS Dasatinib
F
Normalised Isobologram
Das
MK0646

Fig. S2: Combination of MK0646/fulvestrant or MK0646/dasatinib didn't show significant synergistic interactions in LTED cells.
LTED MCF-7 cells were treated with fulvestrant or MK0646 alone in variable concentrations or combination with a fixed dose (Ful 2 nM or MK0646 100 □g/ml) for 48 hours. Growth inhibition was determined using the CellTiter-Blue viability assay as described in Materials and Methods and Figure 3 legend. Dose response curve and synergy analyses were generated in MCF-7/LTED cells (A) and in HCC1428/LTED cells (B) using CalcuSyn Dose Effect Analyzer (detail see Materials and Methods and Figure 3 legend). MCF-7/LTED and HCC1428/LTED cells were treated with dasatinib alone in variable concentrations as indicated or addition of fulvastrant (Ful, 2nM) and/or MK0646 (MK, 100 □g/ml) for 48 hours. Growt [illegible]
HCC1428/LTED cells (D) was determined (ANOVA). Synergy analyses on dasatinib and MK0646 were generated in (detail see Materials and Methods) in MCF7/LTED cells (E) and HCC1428/LTED cells (F).

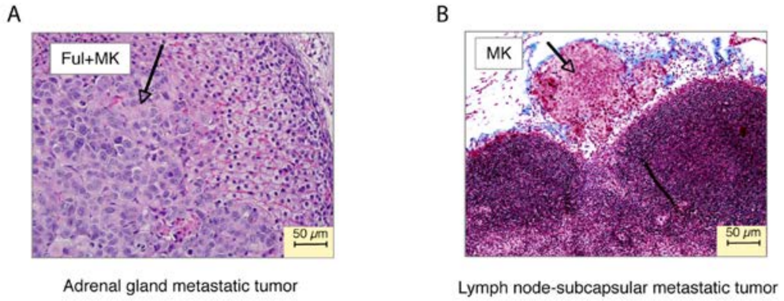

H&E for histologic images showing the metastatic diseases from combination of MK 0646/Fulvestrant (A) or MK0646 monotherapy (B).

Fig. S3: Metastatic diseases in mice with MCF-7/LTED xenografts.

A. A metastatic adrenal gland tumor in the fulvestrant/MK0646 group. B. A metastatic lymph node-subcapsular tumor in the MK0646 alone group.

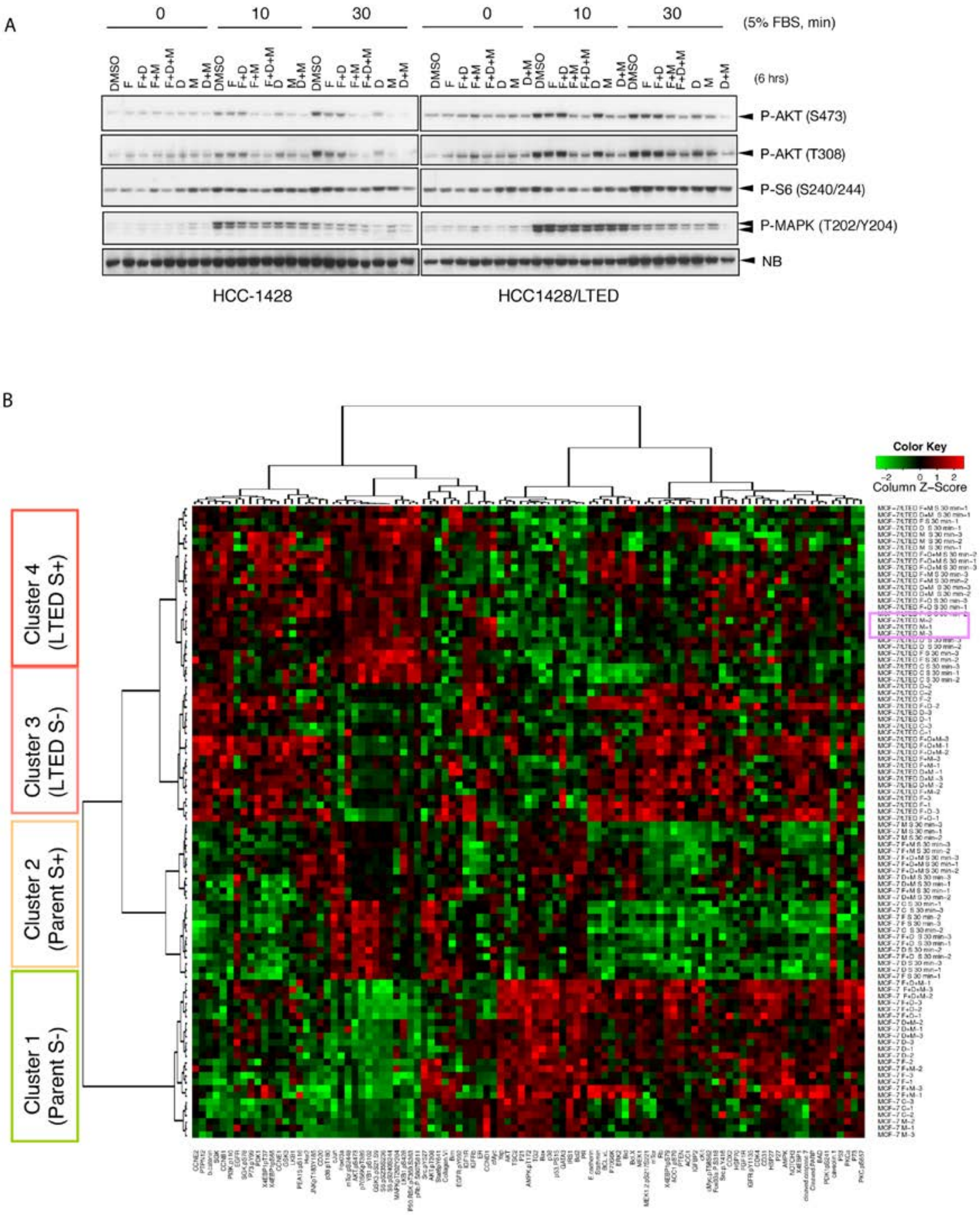

Fig. S4: Signaling response to fulvestrant, dasatinib and MK0646 in LTED cells.
A. LTED HCC1428 cells were treatment with fulvestrant (2 nM), dasatinib (20 nM), MK0646 (100 µg/ml) or combinations as indicated for 6 hours, following by stimulation with or without 5% FBS for variable time as indicated. The cell lysates were used for Western blot (detail see Materials and Methods and the legend of Fig. 6A).
B. LTED MCF-7 cells were starved for overnight, followed by treatment with fulvestrant (2 nM), dasatinib (20 nM), MK0646 (100 µg/ml) or combinations as indicated for 6

hours, following by stimulation with or without 5% FBS for 30 minutes. The cell lysates were analyzed by RPPA. Data are presented in a matrix format: each row represents an antibody target, and each column a sample. In each sample, the ratio of the abundance of the molecule to its median abundance across all samples is represented by the color of the corresponding cell in the matrix (see scale, for expression levels).

Table S1 Sample list in clusters of Supplementary Figure S4B

Cluster 1 (Parental cells, FBS -)	Cluster 2 (Parental cells, FBS 30 min)	Cluster 3 (LTED cells, FBS -)	Cluster 4 LTED cells, FBS 30 min)
MCF-7 C-1	MCF-7 C FBS 30 min-1	MCF-7/LTED C-1	MCF-7/LTED D FBS 30 min-1
MCF-7 C-2	MCF-7 C FBS 30 min-2	MCF-7/LTED C-2	MCF-7/LTED D FBS 30 min-2
MCF-7 C-3	MCF-7 C FBS 30 min-3	MCF-7/LTED C-3	MCF-7/LTED D FBS 30 min-3
MCF-7 F-1	MCF-7 F FBS 30 min-1	MCF-7/LTED F-1	MCF-7/LTED M FBS 30 min-1
MCF-7 F-2	MCF-7 F FBS 30 min-2	MCF-7/LTED F-2	MCF-7/LTED M FBS 30 min-2
MCF-7 F-3	MCF-7 F FBS 30 min-3	MCF-7/LTED F-3	MCF-7/LTED M FBS 30 min-3
MCF-7 F+D-1	MCF-7 F+D FBS 30 min-1	MCF-7/LTED F+D-1	MCF-7/LTED F+D+M FBS 30 min-1
MCF-7 F+D-2	MCF-7 F+D FBS 30 min-2	MCF-7/LTED F+D-2	MCF-7/LTED F+D+M FBS 30 min-2
MCF-7 F+D-3	MCF-7 F+D FBS 30 min-3	MCF-7/LTED F+D-3	MCF-7/LTED F+D+M FBS 30 min-3
MCF-7 F+M-1	MCF-7 F+M FBS 30 min-1	MCF-7/LTED F+M-1	MCF-7/LTED F+M FBS 30 min-1
MCF-7 F+M-2	MCF-7 F+M FBS 30 min-2	MCF-7/LTED F+M-2	MCF-7/LTED F+M FBS 30 min-2
MCF-7 F+M-3	MCF-7 F+M FBS 30 min-3	MCF-7/LTED F+M-3	MCF-7/LTED F+M FBS 30 min-3
MCF-7 F+D+M-1	MCF-7 F+D+M FBS 30 min-1	MCF-7/LTED F+D+M-1	MCF-7/LTED F+D FBS 30 min-1
MCF-7 F+D+M-2	MCF-7 F+D+M FBS 30 min-2	MCF-7/LTED F+D+M-2	MCF-7/LTED F+D FBS 30 min-2
MCF-7 F+D+M-3	MCF-7 F+D+M FBS 30 min-3	MCF-7/LTED F+D+M-3	MCF-7/LTED F+D FBS 30 min-3
MCF-7 D-1	MCF-7 D FBS 30 min-1	MCF-7/LTED D-1	MCF-7/LTED M-1
MCF-7 D-2	MCF-7 D FBS 30 min-2	MCF-7/LTED D-2	MCF-7/LTED M-2
MCF-7 D-3	MCF-7 D FBS 30 min-3	MCF-7/LTED D-3	MCF-7/LTED M-3
MCF-7 M-1	MCF-7 M FBS 30 min-1	MCF-7/LTED D+M -1	MCF-7/LTED D FBS 30 min-1
MCF-7 M-2	MCF-7 M FBS 30 min-2	MCF-7/LTED D+M -2	MCF-7/LTED D FBS 30 min-2
MCF-7 M-3	MCF-7 M FBS 30 min-3	MCF-7/LTED D+M -3	MCF-7/LTED D FBS 30 min-3
MCF-7 D+M-1	MCF-7 D+M FBS 30 min-1		MCF-7/LTED F FBS 30 min-1
MCF-7 D+M-2	MCF-7 D+M FBS 30 min-2		MCF-7/LTED F FBS 30 min-2
MCF-7 D+M-3	MCF-7 D+M FBS 30 min-3		MCF-7/LTED F FBS 30 min-3
			MCF-7/LTED C S 30 min-1
			MCF-7/LTED C S 30 min-2
			MCF-7/LTED C S 30 min-3

Table S2 Antibodies used for RPPA

Antibody	Source	Antibody	Source
4EBP1	Cell Signaling[1]	HSP70	Cell Signaling[1]
4EBP1pS65	Cell Signaling[1]	IGF1R	Cell Signaling[1]
4EBP1pT37	Cell Signaling[1]	IGFR pY1135/Y1136	Cell Signaling[1]
ACC1 pS79	Cell Signaling[1]	IGFBP2	Cell Signaling[1]
ACC1	Epitomics[3]	IGFRb	Cell Signaling[1]
AMPK pT172	Cell Signaling[1]	IRS1	Santa Cruz[2]
AMPK	Cell Signaling[1]	JNK pT183/Y185	Cell Signaling[1]
AKT	Cell Signaling[1]	LKB1 pS428	Cell Signaling[1]
AKT pT308	Cell Signaling[1]	LKB1	Abcam[7]
AKT pS473	Cell Signaling[1]	MAPK pT202/Y204	Cell Signaling[1]
BAD	Epitomics[3]	MEK1/2 pS217/221	Cell Signaling[1]
Bax	Cell Signaling[1]	MEK1	Epitomics[3]
Bcl-X	Epitomics[3]	mTOR pS2448	Cell Signaling1
BCL-xL	Cell Signaling[1]	mTor	Cell Signaling1
β-Catenin	Cell Signaling[1]	NOTCH3	Santa Cruz[2]
Bcl-2	Dako[4]	P21	Santa Cruz[2]
Bid	Epitomics[3]	P27	Santa Cruz[2]
Bim	Epitomics[3]	P38	Cell Signaling[1]
Caveolin 1	Cell Signaling[1]	P38 pT180/Y182	Cell Signaling[1]
Cyclin B1	Epitomics[3]	P53	Cell Signaling[1]
Cyclin D1	Santa Cruz[2]	P53 pS15	Cell Signaling[1]
Cyclin E1	Santa Cruz[2]	P70S6K pT389	Cell Signaling[1]
Cyclin E2	Epitomics[3]	P70S6K	Epitmics[3]
CD20	Epitomics[3]	P73	Cell Signaling[1]
CD31	Dako[4]	P73 pY99	Cell Signaling[1]
CDK4	Cell Signaling[1]	P90 RSK pT359/S363	Cell Signaling[1]
c-Jun	Cell Signaling[1]	PTEN	Cell Signaling[1]
c-Jun pS73	Cell Signaling[1]	PDK1pS241	Cell Signaling[1]
cKit	Epitomics[3]	PDK1	Cell Signaling[1]
c-Myc	Cell Signaling[1]	PEA15 pS116	Invitrogen[8]
c-Myc.pT58/S62	Cell Signaling[1]	PI3K p110	Epitomics[3]
Cleaved caspase 7	Cell Signaling[1]	PKCα pS567	Upstate[9]
Cleaved PARP	Cell Signaling[1]	PKCα	Upstate[9]
Collagen VI	Santa Cruz[2]	PR	Epitomics[3]
COX-2	Cell Signaling[1]	PTPN12	Sigma[10]
E-Cadherin	Cell Signaling[1]	Rb pS807/811	Cell Signaling[1]
EGFR	Santa Cruz[2]	Rb	Cell Signaling[1]
EGFR pY992	Cell Signaling[1]	S6 pS235/236	Cell Signaling[1]
ERK2	Santa Cruz[2]	S6 pS240/244	Cell Signaling[1]
ERα	Lab Vision[5]	SGK pS78	Cell Signaling[1]
ERα pS118	Epitomics[3]	SGK	Cell Signaling[1]
FGF1R	Santa Cruz[2]	Src pY416	Cell Signaling[1]
Fox03a pS318	Cell Signaling[1]	Src pY527	Cell Signaling[1]
Fox03a	Cell Signaling[1]	Stat6 pY641	Cell Signaling[1]
GATA3	BD Biosciences[6]	Stathmin	Epitomics[3]
GSK-3	Santa Cruz[2]	TG2	Abcam[7]
GSK-3α/β pS21/9	Cell Signaling[1]	TSC2	Epitomics[3]
Her2	Cell Signaling[1]	Yap	Cell Signaling[1]
HSP27	Cell Signaling[1]	YB1 pS102	Cell Signaling[1]

[1] Beverly, MA; [2] Santa Cruz, CA; [3] Burlingame, CA; [4]Carpinteria, CA; [5] Fremont, CA; [6] San Jose, CA; Abcam, Cambridge, MA; [8]Carlsbad, CA; [9] Billerica, MA; [10]Louis, MO
